# Single-best Choice Between Intermittent Versus Continuous Renal Replacement Therapy: A Review

**DOI:** 10.7759/cureus.5558

**Published:** 2019-09-03

**Authors:** Nida Fathima, Tooba Kashif, Rajesh Naidu Janapala, Joseph S Jayaraj, Aisha Qaseem

**Affiliations:** 1 Internal Medicine, Sri Siddhartha Medical College, Tumkur, IND; 2 Cardiology, Heart and Vascular Institute, Dearborn, USA; 3 Internal Medicine, Icahn School of Medicine at Mount Sinai, Queens Hospital Center, New York, USA; 4 Internal Medicine, Emory Johns Creek Hospital, Georgia, USA

**Keywords:** acute kidney injury, renal failure, renal replacement therapy, intermittent hemodialysis, continuous renal replacement therapy, renal recovery, effectiveness

## Abstract

Critically ill patients often develop multiorgan dysfunction syndrome. Acute kidney injury (AKI) is part of it. Renal replacement therapy (RRT) remains the primary choice of treatment in severely ill patients who develop AKI. Recent data have shown increased use of RRT in AKI patients. Therefore, the right choice of RRT plays an important role in the renal recovery of such patients. The question of which mode of RRT to apply has been the topic of study in the last two decades. Whether RRT should be conducted in intermittent mode, as intermittent hemodialysis (IHD), or in continuous mode, as continuous renal replacement therapy (CRRT), is still being investigated. CRRT has a hypothetical advantage when compared to IHD, as it involves a process in which there is gradual removal of fluids, better control of urea, better maintenance of the acid/base balance, and hemodynamic stability. However, IHD is more practical, cost-effective, does not require anticoagulation, decreases the bleeding risk, and removes the solute efficiently and rapidly in acute life-threatening conditions.

Other modalities of RRT like sustained low-efficiency daily dialysis (SLEDD) and prolonged intermittent renal replacement therapy (PIRRT) have shown to encompass the benefits of both CRRT in terms of hemodynamic stability and IHD in terms of cost-efficiency. Although SLEDD is progressively being used as an alternative to CRRT and IHD, very few studies have shown to support it. In this article, we try to summarize the advantages and disadvantages of the different techniques used in RRT. With SLEDD gaining more popularity among the different modalities of RRT, we want to assess the possibility of its routine implementation as the single-best choice for RRT.

## Introduction and background

Renal replacement therapy (RRT) is still the core treatment in critically ill patients and those who develop severe acute kidney injury (AKI) [1]. The most common complication in severely ill patients is acute renal failure (ARF) with an approximately 50% or greater mortality rate. About 5%-10% of these critically ill and unstable patients require RRT as their primary treatment [2]. RRT is used to replace the kidney functions in both acute and chronic renal failure. It helps maintain the mean arterial pressure, cerebrovascular perfusion, and renal perfusion and helps attain a hemodynamic environment in severely ill patients. Among the patients requiring RRT who survive the critical phase of their illness, the majority will be free of RRT at the time of their discharge. The technique involved in all modes of RRT is the removal of water and solutes and the exchange of plasma using dialysis and filters. Each type of RRT may use processes like filtration, ultrafiltration, and diffusion. In the filtration process, the solute is exchanged through a permeable membrane and waste products are discarded. Ultrafiltration is a process in which water is transported across a semi-permeable membrane while solutes are transported across the membrane by diffusion in the diffusion and convection methods.

Due to an impaired filtration process through the renal system and subsequent volume overload in critically ill patients, patients require the daily removal of fluid and electrolytes from the body, which can be achieved by either intermittent hemodialysis (IHD) or continuous renal replacement therapy (CRRT). Newer modes involved are prolonged intermittent renal replacement therapy (PRRT) and sustained low-efficiency daily dialysis (SLEDD) [3]. Peritoneal dialysis (PD) is also an infamous mode of RRT and is rarely used. The subtypes of CRRT are hemofiltration, hemodialysis, and hemodiafiltration. When comparing IHD and CRRT, it has been studied that IHD often exacerbates hemodynamic instability with a high rate of fluid and solute removal, whereas CRRT involves the slow and constant removal of water and solutes from the plasma and is preferred for managing unstable patients [4]. CRRT has shown to be better in terms of renal recovery outcome than IHD in patients with AKI or critically ill patients who end up developing AKI. However, their efficacy and superiority have not been proven [1]. The CRRT technique has some limitations, which need to be reviewed before making the right choice of RRT. SLEDD is an emerging mode, which is receiving a lot of appreciation and has been accepted as an alternative therapy to CRRT in hemodynamically unstable patients who develop AKI.

## Review

Different modalities of RRT and role of CRRT

**Figure 1:**
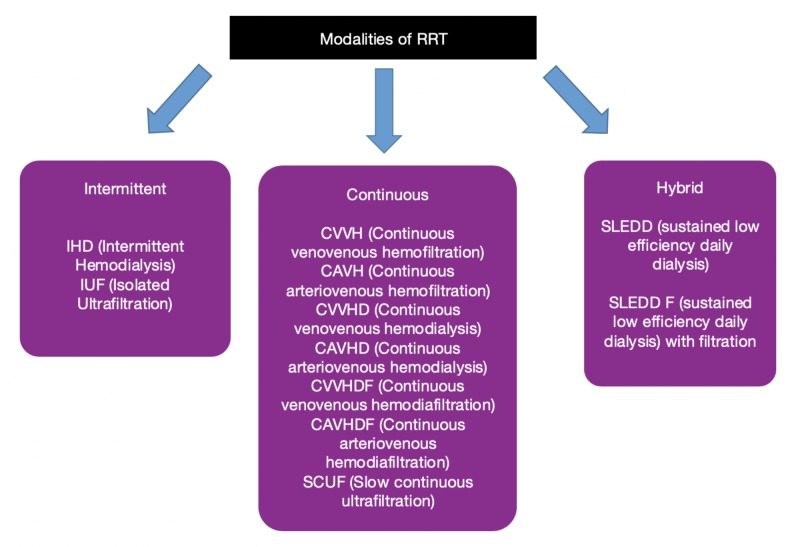
Different modes of RRT RRT - Renal Replacement Therapy, IHD - Intermittent Hemodialysis, IUF - Isolated Ultrafiltration, CVVH - Continuous Venovenus Hemofiltration, CAVH - Continuous Arteriovenous Hemofiltration, CVVHD - Continuous Venovenous Hemodialysis, CAVHD - Continuous Arteriovenous Hemodialysis, CVVHDF - Continuous Venovenus Hemodiafiltration, CAVHDF - Continuous Arteriovenous Hemodiafiltration, SCUF - Slow Continuous Ultrafiltration, SLEDD - Sustained Low Efficiency Daily Dialysis, SLEDD F - Sustained Low Efficiency Daily Dialysis with Filtration

Nearly 70% of patients with AKI require RRT. An ideal RRT is one that improves uremia through toxin clearance, maintains adequate fluid volume, corrects acid-base abnormalities, and helps promote renal function recovery. Continuous renal replacement therapy (CRRT) and intermittent hemodialysis (IHD) are the main types of RRT used in the management of AKI and severely ill patients. Sustained low-efficiency dialysis (SLEDD) is a hybrid mode of RRT. Figure 1 shows the types of RRT and Table 1 compares its various modes. It is observed that CRRT and SLEDD tend to be significantly used in patients with hemodynamic instability. Likely, there is considerable variability as to how each of these types of RRT is utilized and prescribed. CRRT involves the following techniques: continuous arteriovenous hemofiltration (CAVH), continuous venovenous hemofiltration (CVVH), continuous arteriovenous hemodialysis (CAVD), continuous venovenous hemodialysis (CVVHD), and hemodiafiltration.

**Table 1:** Comparison between different modes of RRT RRT - Renal Replacement Therapy, IHD - Intermittent Hemodialysis, CRRT - Continuous Renal Replacement Therapy, SLEDD - Sustained Low Efficiency Daily Dialysis, CRF - Chronic Renal Failure

MODE OF RRT	IHD	CRRT	SLEDD
Mechanism of solute removal	Intermittent solute removal by diffusion (rapid)	Continuous removal (24hr ultrafiltration) of small to middle molecules diffusion, convection or both.	Slow/sustained low efficiency daily dialysis of small to middle solute molecules by diffusion
Duration	3-4hrs/day	24hrs/day	6-12hrs/day
Dialysate flow rate	>/= 500 ml/min	17-34 ml/min	300 ml/min
Blood flow rate	> 200 ml/min	< 200 ml/min	200 ml/min
Hemodynamic stability	Poor	Good	Fair - Good
Efficiency	High	Low - Moderate	Moderate
Cost	Low	High	High
Anticoagulation	Not needed	Important	Usually not needed
Patient clinical conditions used in	In ambulatory CRF patients, hyperkalemia	Unstable and non-ambulatory critically ill patients, hyperkalemia, uremia, sepsis	Critically ill patients
Complications	Hypotensive episodes	Hypotensive episodes	Air embolism, hypothermia

Critically ill patients usually develop acute kidney injury and acute renal failure, along with multi-system organ failure, and end up requiring the daily administration of large volumes of fluids in the form of nutrition, medications, and blood products. This often leads to fluid overload and disrupts the acid/base status of the patient. Initially, such patients were managed with the traditional technique of RRT, which is IHD [1,5]. Later, in the 1980s, Kramer et al. introduced CRRT, which allows blood purification 24 hours per day, as an alternative to the traditional IHD [6]. CRRT initially used a simple technique of hemofiltration CAVH without any pumps. However, this concept often lacked efficiency, hence new improvements to the technique incorporated blood pumps, which is known as CVVH. CRRT involves a continuous technique in which solute (ultra-filtrate) is removed through large pores and is replaced by substitute fluid. Later, the diffusion method was also implied in CRRT by introducing additional pumps to the machines. CRRT has brought greater control of urea, better electrolyte balance, helped maintain the acid/base status, and enhanced hemodynamic stability as compared to the traditional IHD mode [7].

Aspects to keep in mind before choosing the type of RRT (CRRT vs. IHD)

Hemodynamic Stability

Despite the uncertainty, fluid-overloaded patients were more likely to benefit from CRRT vs. traditional IHD. CRRT has been suggested to offer better efficiency in the administration of nutritional fluids [8]. The hemodynamic advantage in CRRT seemed to be due to hypothermia, which improved venous return and blood pressure. Augustine et al. showed a significant difference between IHD and CRRT [9]. Table 1 summarizes the different modes of RRT and their use in terms of patients' hemodynamics. These hemodynamic advantages of CRRT over IHD are, however, not confirmed in a prospective study [10].

Solute Removal and Duration

IHD involves a faster rate of fluid and solute removal (>/= 500 ml/min); this rapid rate often exacerbates hemodynamic instability. With the higher intensity of solute removal, there is an associated significant removal of the drug as well, which can, in turn, make them subtherapeutic, lead to their inadequacy, and cause electrolyte imbalances [11]. CRRT includes the slow and continuous removal of water and solutes from the plasma (17-34 ml/min) and is currently preferred for managing hemodynamically unstable patients [7,12-13]. Table 1 shows the difference in blood flow rate and dialysate flow rate among IHD and CRRT. Decreased blood and dialysate flow rate and prolonged duration have shown to be better in the removal of solute from extra-plasmatic compartments due to better mobilization of solutes. One of the disadvantages of the CRRT process, as opposed to IHD, is that filters get clotted frequently and thus there is a regular need to change the filters for adequate filtration. The removal of cytokines and other inflammatory markers, such as IL4, TNF alpha, IL10, or IL8, was significantly better with CRRT as compared to IHD. Although, in acute life-threatening conditions like AKI with hyperkalemia, rhabdomyolysis, and poisoning, the rapid removal of solutes is required, for which IHD is the choice of RRT and is readily used for managing such patients. The average duration of treatment with CRRT was reported to be 19.5 hours per day and as low as 13.4 hours per day observed individual value [7,14].

Renal Recovery

One of the primary concerns in AKI patients is renal recovery. With each hypotensive episode, the GFR decreases, causing ischemic injury to the kidney and delays the recovery of renal function [15]. IHD causes a larger number of hypotensive episodes and thus, theoretically, it might slow down the recovery process, resulting in patients requiring chronic dialysis and increasing the mortality rate. CRRT, due to its principle (24 hours/day) of continuous exchange of fluids, has been better at maintaining homeostasis in unstable patients, thus improving recovery of renal functions and lowering the mortality rates. The difference between renal recovery due to IHD and CRRT from different studies is shown in Table 2. A study by Schneider et al. adds to the growing evidence to suggest that there is an improved likelihood for renal function recovery in critically ill survivors of AKI with continuous modalities of RRT [16]. However, not all studies have demonstrated the superiority of CRRT in this regard [7,9-11,13].

**Table 2:** Renal recovery outcomes between IHD and CRRT IHD - Intermittent Hemodialysis, CRRT - Continuous Renal Replacement Therapy

Study	Sample Size		Percentage of Dialysis Dependence at the Time of Discharge	
	CRRT	IHD	CRRT	IHD
Mehta et al. [13]	84	82	14.0	7.0
Vinsonneau et al. [11]	175	184	1.8	0.0
Uehlinger et al. [10]	70	55	2.7	3.7
Augustine et al. [9]	40	40	61.5	66.7

Patient Conditions Benefiting from Different RRT

Although controversial, CRRT use in severely volume overloaded patients can be defended. Other conditions where CRRT can be used are combined acute renal and hepatic failure [17], intracranial trauma patients, cerebral edema, and lithium toxicity because of its better hemodynamic stability in such patients [18]. Table 3 shows the clinical scenario and the choice of RRT. Compared to IHD, CRRT can be more efficient in patients with sepsis-induced AKI in removing excess water and metabolic waste and lowers the levels of pro-inflammatory cytokines, maintains homeostasis, lowers adverse effects on the cardiovascular system, and significantly improves the prognosis of the patient. It also shortens the time for organ support needs and the duration of intensive care unit (ICU) stay [4,7]. IHD is favored in patients with bleeding risk, acute hyperkalemia, and rhabdomyolysis.

**Table 3:** Choice of RRT in a given clinical scenario RRT- Renal Replacement therapy, SLEDD - Sustained Low Efficiency Daily Dialysis, CRRT - Continuous Renal Replacement Therapy, IHD - Intermittent hemodialysis, PIRRT - Prolonged Intermittent Renal Replacement Therapy, PD - Peritoneal Dialysis

	Hemodynamic instability	Fluid Overload	Acute Intracranial Injury/Hypertension	Life-Threatening Conditions
First Option	CRRT/SLEDD/PIRRT	CRRT/SLEDD/PIRRT	CRRT/PD	IHD
Second Option	PD	IHD	PIRRT	PIRRT
Third Option	IHD	PD	IHD	CRRT
Fourth Option	-	-	-	PD

Survival Benefit

Many studies and randomized controlled trials (RCTs) have suggested no advantage in the survival outcome with CRRT when compared to IHD. Few studies have demonstrated more favorable hemodynamic stability with CRRT, but it is not clearly better than IHD in the survival outcome [9-11,13]. The findings of trials by Mehta et al. etc. are shown in Table 4. It showed higher ICU mortality in patients treated with CRRT when compared to IHD (59.5 vs. 41.5%) [13]. However, these findings were limited by apparent baseline imbalances between the groups; patients randomized to the CRRT group had greater severity of illness. There was no difference between the groups in terms of renal recovery [7,13]. Other studies showed that CRRT was better or no different than IHD in terms of mortality.

**Table 4:** Mortality outcome between patients treated with IHD vs CRRT IHD - Intermittent Hemodialysis, CRRT - Continuous Renal Replacement Therapy

Study	Primary Outcome	Sample Size		Mortality (%)	
		CRRT	IHD	CRRT	IHD
Mehta et al. [13]	ICU mortality	84	82	59.5	41.5
Vinsonneau et al. [11]	In-hospital mortality	175	184	67.4	68.5
Uehlinger et al. [10]	In-hospital mortality	70	55	47.0	51.0
Augustine et al. [9]	In-hospital mortality	40	40	67.5	70.0

Cost Efficiency

Cost should be kept in mind before choosing the type of RRT. IHD is more cost-effective than CRRT [19]. The fluid replacement cost, need for filter replacement, dialysate fluid cost, and extracorporeal circuit expenses are higher in CRRT globally. The median difference in price was US$ 289.6 per day, which did not favor CRRT over IHD [20]. Overall, all studies showed a cost advantage with IHD when compared to CRRT. Due to the continuous procedure involved in CRRT, it requires more extended patient immobilization. High in-patient admissions and the need for specialized machines, trained nurses, technicians, and staff further adds to the economic burden of CRRT.

Anticoagulation Need

Because of the continuous filtration process (24 hours/day) and more extended periods of immobilization in CRRT, patients will require anticoagulation, which will increase their bleeding risk, especially in patients who recently had surgery, trauma, or have medical conditions prone to bleeding. To overcome this drawback, citrate anticoagulation is used in CRRT. Its effects might be beneficial, as it is an easily applicable process and has excellent diffusive clearance properties [1]. Another drawback of CRRT was the clotting of filters because it is a continuous process. A study, however, showed that patients would be better treated with IHD vs. CRRT, as IHD therapy does not require anticoagulation [11].

Alternative to CRRT and IHD

Sustained/Slow Low-Efficiency Daily Dialysis (SLEDD)

SLEDD is an alternate mode of RRT. In this technique, intermittent dialysis is applied at a slower rate and over a prolonged period of time (>6 hours/day). Its main advantage is its flexibility in terms of intensity and duration. The filtration rate can be adjusted as per the needs of the patient. Observational data from a single-center suggest that SLEDD is a reasonable mode of RRT, which is adequate, hemodynamically well-tolerated, potentially anti-coagulation-free, and possibly cost-effective [19]. With respect to CRRT, only two small RCTs have compared SLEDD and CRRT [12,21]. Using invasive monitoring, these authors found no significant differences in all measured hemodynamic parameters (mean arterial pressure, systemic vascular resistance, and cardiac output) between SLEDD and CRRT. They also identified that they have a similar urea and creatinine clearance rate. Table 5 shows a brief comparison between SLEDD and CRRT. A smaller study randomized 16 patients to receive three sessions with either CRRT or SLEDD (with an added hemofiltration component), which showed that both fluid removal and hemodynamic parameters were similar in both groups [12]. Although these preliminary data suggest that SLEDD may be used as an alternative to CRRT, further studies that utilize patient-relevant outcomes are required to support and make precise the role of SLEDD. A newer SLEDD strategy has recently been outlined in which the CRRT treatment concept was applied over a shortened time period of nine hours, increasing the blood and dialysate flow rates. This, accelerated venovenous hemofiltration (AVVH) modality retains many of the possible advantages of SLEDD, but dedicated commercial solutions were still required. A retrospective case series demonstrated the adequate removal of solute, better hemodynamic tolerability, and the ability to avoid anticoagulation use with SLEDD [22].

**Table 5:** Comparison between SLEDD and CRRT SLEDD - Sustained Low Efficiency Daily Dialysis, CRRT - Continuous Renal Replacement Therapy

SLEDD	CRRT
Higher fluid shifts	Hemodynamic stability, reduced risk for cerebral edema
Require conventional equipment, simple procedure.	Require more complex equipment.
Easy to perform, patient mobility, flexible timings 12hrs/day.	Require skilled and trained technical personnel. Patient immobilization needed.
Lower cost	Higher cost
Anticoagulation with heparin if needed.	Anticoagulation required with heparin and citrate.
Less risk of bleeding complication, no bag handling -> less risk of infection	High risk of bleeding complications.
Not so popular, risk of hypophosphatemia	Superior in solute removal and volume control, adequate nutritional support is possible.

Prolonged Intermittent Renal Replacement Therapy (PIRRT)

PIRRT is another alternative that has the benefits of both CRRT, in terms of hemodynamic stability, and IHD, in terms of cost-effectiveness [23-24]. The Ratanarat et al. study aimed to assess PIRRT in the aspects of efficiency and hemodynamic outcomes [23]. In a case report that compared the three types of RRT used for severe acute lithium toxicity, PIRRT was the first to be used. A lithium level of 13.2 mmol/L was recorded after a 51-year-old man attempted suicide. He was treated with IHD, PIRRT, and CVVH after more than 55 hours, lithium clearance was comparable between IHD and PIRRT [18]. Both of these types were superior to CVVH. The efficacy of therapy with a daily session with PIRRT was higher than IHD or CVVH. PIRRT was shown to have been a useful strategy and an acceptable form of dialysis among patients with acute lithium intoxication.

Peritoneal Dialysis (PD)

PD is a rare type of RRT. It can be especially helpful in hemodynamically unstable and fragile patients; it is an ignored modality used in AKI. It can be used in patients with higher bleeding risk and in children [22], especially neonates and small children with post-cardiac surgery AKI and hemolytic uremic syndrome (HUS) [7]. In a Vietnamese study of infected patients, continuous hemofiltration was superior to PD [25]. The other studies showed no differences [22]. The PD modality was unlikely to be sufficient to obtain satisfactory solute clearance as compared to the other modalities of RRT.

## Conclusions

RRT treatment efficiently manages critically ill patients with AKI. It helps decrease the mortality rate among such patients. IHD treatment may delay the renal recovery process in critically ill and unstable patients when compared to CRRT treatment. However, it is cost-efficient and the treatment of choice for managing acute patient conditions. CRRT has shown to be more efficient as compared to IHD in maintaining homeostasis, removing the excess volume of fluids and metabolic waste and significantly improve the prognosis of volume overloaded patients. Like IHD, CRRT also has its limitations such as prolonged immobilization, higher cost, availability of trained technicians, resources, need for anticoagulation, and a requirement for in-patient admissions. Despite these limitations, it is still considered superior to IHD while treating unstable patients. Its choice of treatment in stable patients is controversial. In terms of survival outcome benefit, no study has significantly proven the superiority of CRRT when compared to IHD. Newer techniques like SLEDD and PIRRT exhibit the benefits of both CRRT in terms of hemodynamic stability and IHD in terms of cost-effectiveness. Recently, SLEDD has been considered an acceptable alternative to CRRT in critically ill patients with AKI, but only a few studies have supported it. With the never-ending debate for the best choice of RRT between IHD vs CRRT, it is seen that the SLEDD technique is being more appreciated. We need more research to prove the superiority of SLEDD and assess its possibility for routine implementation as the single-best therapy for RRT.
